# Spectral domain OCT features in type 2 macular telangiectasia (type 2 MacTel): its relevance with clinical staging and visual acuity

**DOI:** 10.1186/s40942-022-00378-0

**Published:** 2022-04-05

**Authors:** Ramesh Venkatesh, Nikitha Gurram Reddy, Pranjal Mishra, Sameeksha Agrawal, Deepashri Mutalik, Naresh Kumar Yadav, Jay Chhablani

**Affiliations:** 1 Department of Retina and Vitreous, Narayana Nethralaya, #121/C, 1st R Block, Chord Road, Rajaji Nagar, Bengaluru, 560010 Karnataka India; 2 School of Medicine, Medical Retina and Vitreoretinal Surgery, University of Pittsburgh, 203 Lothrop Street, Suite 800, Pittsburg, PA 15213 USA

**Keywords:** Type 2 MacTel, Optical coherence tomography, Visual acuity, Clinicals stages, Macular telangiectasias

## Abstract

**Background:**

To report spectral domain optical coherence tomography (SDOCT) imaging findings in type 2 macular telangiectasia (MacTel) and correlate them with clinical stages and visual acuity.

**Methods:**

This retrospective, cross-sectional study included type 2 MacTel cases who underwent SDOCT imaging with Spectralis machine. Macular SDOCT images were analysed. Imaging features were tested for correlation with different clinicals stages and visual acuity.

**Results:**

212 eyes of 108 type 2 MacTel patients were included. Hyperreflective middle retinal layer (87%) was the most frequently detected abnormality. This was followed by inner retinal cavities (49%), outward bending of inner retinal layers (35%), retinal pigment clumps (35%) and foveal contour irregularity (31%). Hyperreflective middle retinal layers (p < 0.001), inner (p = 0.032) and outer retinal (p = 0.002) cavities and internal limiting membrane drape (p = 0.031) were associated with poor vision in non-proliferative group and presence of retinal pigment clumps (p = 0.002), subretinal fluid (p = 0.037) and foveal contour irregularity (p < 0.001) were associated with poor vision in proliferative group.

**Conclusion:**

The described SDOCT features are practical for the diagnosis and staging in type 2 MacTel. Presence of hyperreflective middle retinal layers, hyporeflective inner and outer retinal cavities and internal limiting membrane drape were associated with poor vision in the non-proliferative group while retinal pigment clumps and subretinal neovascular membrane were associated with proliferative group and poor vision. Further long-term studies are required to describe the progressive and sequential changes on SDOCT.

## Background

The term “type 2 macular telangiectasia” (type 2 MacTel) has been classically described as an idiopathic, non-familial, bilateral disease of the elderly affecting the macular Müller cells and capillary network, associated with changes in the inner and outer retinal structure and ultimately leading to development of abnormal neovascular complexes [1]. In 1993, based on the clinical and angiographic findings, Gass and Blodi provided additional clinical staging of type 2 MacTel into five stages beginning with perifoveal greying and loss of retinal transparency as stage 1 to no visible/occult telangiectasias, dilated right angled venules, retinal pigment clumps (RPC) as stages 2, 3 and 4 respectively and development of subretinal neovascular membrane (SRNVM) as stage 5 [2]. Yannuzzi et al. simplified the classification proposed by Gass and Blodi into two distinct stages: non-proliferative and proliferative [3]. With the availability of various imaging modalities, a better understanding of the clinical features and natural history of the disease and intrinsic pathogenetic mechanisms was provided by the Mactel Study Project from 2005 [4].

Spectral-domain optical coherence tomography (SDOCT), a non-invasive imaging modality allows high-speed acquisition of macular retinal scans with high resolution. Thus, the SDOCT has become an invaluable imaging tool for diagnosing and studying type 2 MacTel. There are a few articles which have been published in literature describing the SDOCT imaging features such as foveal floor flattening, internal limiting membrane (ILM) drape, degenerative inner and outer retinal hyporeflective cavities, disruption of the external limiting membrane, ellipsoid zone and interdigitation zone and presence of macular hole and SRNVM in type 2 Mactel and its relationship with visual acuity [5–8]. However, none of the studies make a link between previous staging based on fundus photograph and OCT. Hence, more studies are required to establish an association between the clinical features, SDOCT features and the disease pathogenesis in type 2 MacTel.

The purpose of the current study was to describe the SDOCT imaging features, their relationship with the different clinical stages on fundus examination and visual acuity and further to establish a connection between the SDOCT findings and disease pathogenesis in a large cohort of type 2 MacTel cases.

## Methods

In this retrospective observational study, we reviewed the clinical records and SDOCT images of patients diagnosed with type 2 MacTel attending the retina services at a super speciality eye hospital between January 2011 and December 2020. The study complied with the tenets of the Declaration of Helsinki and was approved by the local Institutional Review Board/Ethics Committee. Because the study was a retrospective analysis, waiver for informed consent was obtained.

The diagnosis of type 2 Mactel was made based on a constellation of clinical findings as described by Gass and Blodi [2] and other imaging features using confocal blue reflectance, fluorescein angiography and SDOCT images. Patients with other concomitant macular pathologies or those with confusing diagnosis of type 2 MacTel were excluded from the study. Patients having media opacities which did not allow good quality OCT scans to be acquired for analysis were excluded from the study. Data recording included demographics, Snellen’s best corrected distance visual acuity and SDOCT imaging features at the baseline visit.

**Fig. 1:**
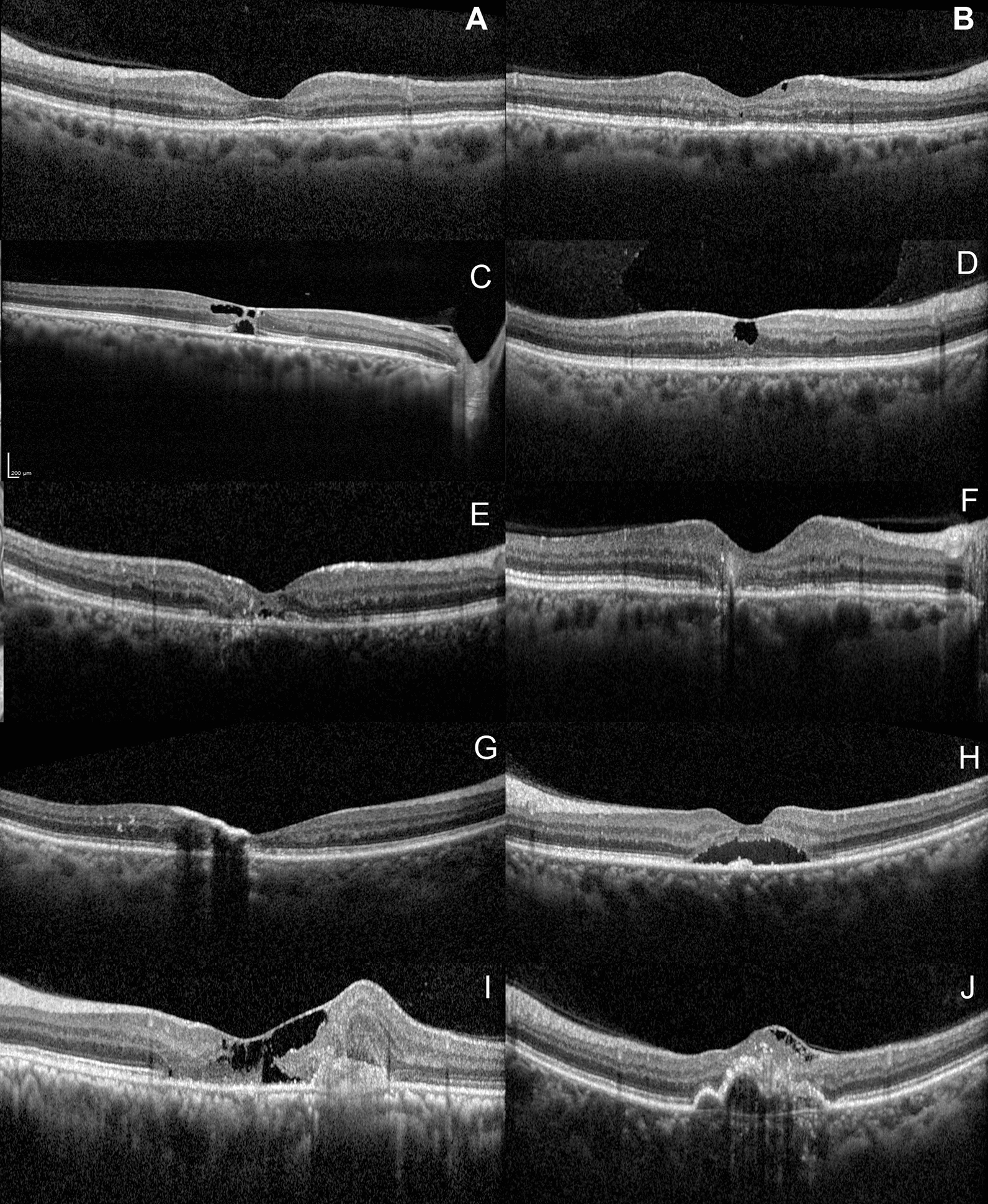
Various abnormalities found in optical coherence tomography images of macular telangiectasia type 2 (MacTel type 2). **A** Increased reflectivity of the inner retina at the middle retinal layers at the temporal parafovea and asymmetric foveal contour are present. **B** Hyperreflective middle retinal layers temporal to the fovea with hyporeflective inner retinal cavity. **C** Inner and outer retinal hyporeflective cavities are noted. **D** Hyporeflective inner retinal cavity can be found at the foveal centre with an overlying ILM drape is present. **E** Superficial retinal crystals are seen as hyperreflective spots in the superficial layer of the retina without any back shadowing and outward turning of the inner retinal layers is noted with shallow sub foveal SRF. **F** Outward turning of the inner retinal layers is noted. **G** Pigment migration to the inner retinal layers with back shadowing is noted. **H** Hyperreflective middle retinal layers with subfoveal SRF is noted. **I** Foveal contour distortion with presence of ILM drape with outward turning of the middle retinal layers and presence of subretinal neovascular membrane is noted. **J** ILM drape with foveal contour irregularity is noted. There is a hyperreflective material noted in the retinal layers breaching the retinal pigment epithelium suggestive of retino-choroidal anastomosis

SDOCT scans were obtained using the Spectralis machine (Spectralis, Heidelberg Engineering, Germany) in all eyes. Macular volumetric assessments consisting of 512 A-scans per line with 30° scanning area and 25-line horizontal raster volume scans centred at the fovea were performed. SDOCT scans having a quality score ≥ 20 were used for analysis and interpretating the findings. All the images encompassing the macular area were analysed by a single masked observer (NR) who was unaware of the clinical findings and the following features were noted from inner retina to outer retina in a sequential manner: (1) irregularity of the foveal contour, (2) ILM drape, (3) hyperreflectivity of the middle retinal layers (MRL) i.e., between the inner plexiform to the outer plexiform layers, (4) identification of retinal crystals as superficial hyperreflective retinal dots, (5) hyporeflective inner retinal cavities, (6) hyporeflective outer retinal cavities, (7) outward bending of inner retinal layers (IRL), (8) hyperreflective RPC with underlying shadowing, (9) subfoveal subretinal fluid (SRF), (10) macular hole either full-thickness or pseudohole or lamellar macular hole, (11) SRNVM, and (12) retino-choroidal anastomosis (RCA) (Fig. 1).

### Definitions of the various SDOCT features

Foveal irregularity was described as asymmetry at the foveal floor either due to the dipping or thickening of IRL. ILM drape was described as the presence of cavitation under the foveal floor with only the ILM in place over these areas. Hyperreflective MRL at the perifoveal region was identified as increased reflectivity and thickening between the inner and outer plexiform layers. Superficial hyperreflective retinal dots on SDOCT were noted as bright hyperreflective dots sitting on the ILM surface with absence of underlying shadowing. Hyporeflective cavity was divided into the inner and outer cavities based on the boundary created by the external limiting membrane (ELM). Hyporeflective cavities located above the ELM were considered as inner retinal cavities while those which were located below the ELM were considered as outer retinal cavities. Outward bending of IRL was identified as the dipping of the IRL onto the outer retina due to the collapse of the inner retinal skeleton. Retinal pigment clumps were noted as hyperreflective clumps in the middle or superficial retinal layers with presence of underlying shadowing. Accumulation of SRF at the fovea was defined as subfoveal SRF and was considered separate from the outer retinal hyporeflective cavities. Identification of an oval, fusiform hyperreflective lesion in the subretinal space above the retinal pigment epithelium (RPE) with or without associated SRF or exudation was defined as SRNVM. Extension of the retinal neovascularisation directly to the underlying choroid following an RPE elevation and breach was defined as RCA.

## Statistical tests

All data were analysed using GraphPad Prism version 9.0.0 (121) for Windows, GraphPad Software, San Diego, California USA, www.graphpad.com. The Shapiro–Wilk normality test was used to test the normality of the data sets. Snellen’s vision data was converted to logMAR vision for statistical analysis. Different SDOCT imaging features were described as numbers and percentages. Quantitative variables between the two groups were analysed using the Mann–Whitney U test for non-parametric data and unpaired t test for parametric data. Chi-square test was used to compare the categorical data between the two groups. Correlations between the different SDOCT biomarkers and visual acuity were analysed using the Spearman’s correlation test. The binary responses of the various SDOCT imaging features were converted to numerical values (0 = absent and 1 = present) for the purpose of studying correlation with visual acuity. Multiple variable linear regression analysis was performed between the visual acuity as the dependent variable and statistically significant SDOCT features on corelation matrix as independent variables. p values < 0.05 were considered statistically significant.

## Results

### Patient demographics

In total, 212 eyes of 108 patients with findings of type 2 MacTel were included for analysis. The mean age at the initial visit was 62.3 ± 9.46 years, and the patients were predominantly female (n = 69, 64%). All patients were of Indian ethnicity. Seventy-five of 108 patients (69%) had a history of diabetes mellitus. The mean logMAR visual acuity of the whole dataset was 0.447 ± 0.334 (Snellen equivalent = 20/56).

Table 1 describes the different SDOCT imaging features in study patients. It also describes and compares the visual acuity in the presence or absence of these findings. Hyperreflective MRL (n = 184, 87%) in the perifoveal region was the most common imaging finding seen on SDOCT. This was followed by presence of hyporeflective inner retinal cavities (49%), outward bending of IRL (35%), presence of hyperreflective RPC (35%) and irregularity of the foveal contour (31%). Presence of sub foveal SRF was noted in 34 (16%) eyes. Of which, 15 of the 34 (44%) eyes had associated SRNVM or RCA. The remaining 19 (56%) eyes showed SRF without any proliferative stage. Macular hole was not seen in any case. Presence of foveal contour irregularity (p < 0.001), hyperreflective MRL (p < 0.001), hyperreflective RPC (p < 0.001), SRNVM (p = 0.001) and RCA (p = 0.045) significantly affected the visual acuity compared to the other imaging features.

**Table 1:** Various SDOCT imaging features and visual acuity changes in patients with type 2 MacTel

Variable	N (%)	Visual acuity when finding present	Visual acuity when finding absent	P value
Irregularity of foveal contour	66 (31)	0.563 ± 0.299	0.361 ± 0.307	< 0.001
Internal limiting membrane drape	62 (29)	0.441 ± 0.365	0.450 ± 0.321	0.572
Hyperreflective middle retinal layers	184 (87)	0.421 ± 0.340	0.621 ± 0.226	< 0.001
Superficial hyperreflective retinal dots	6 (3)	0.623 ± 0.448	0.442 ± 0.330	0.299
Hypo reflective inner retinal cavities	104 (49)	0.423 ± 0.344	0.471 ± 0.323	0.125
Outward bending of inner retinal layers	74 (35)	0.493 ± 0.304	0.423 ± 0.347	0.068
Hypo reflective outer retinal cavities	32 (15)	0.393 ± 0.358	0.457 ± 0.329	0.175
Hyperreflective retinal pigment clumps	74 (35)	0.572 ± 0.336	0.381 ± 0.314	< 0.001
Subfoveal SRF	34 (16)	0.515 ± 0.279	0.435 ± 0.342	0.093
Subretinal neovascular membrane	41 (19)	0.591 ± 0.311	0.413 ± 0.331	0.001
Retino-choroidal anastomosis	19 (9)	0.606 ± 0.390	0.432 ± 0.325	0.045

SRF, subretinal fluid

### OCT features in different clinical stages of type 2 MacTel

Table 2 summarizes the different SDOCT features noted in different clinical stages of type 2A Mactel on retinal examination as defined by Gass and Blodi [2] and by Yannuzzi et al. [3]. The SDOCT features like hyperreflective MRL, hyporeflective inner and outer retinal cavities, ILM drape, outward bending of IRL were commonly noted with the non-proliferative clinical stages of type 2 MacTel. Other SDOCT features like foveal contour irregularity, hyperreflective RPC and presence of SRNVM/RCA were associated with the advanced clinical stages of the disease. In addition, 25 eyes showed a disparity between the clinical staging of the disease on retinal examination and SDOCT features. These eyes were wrongly staged as non-proliferative disease (as per Yannuzzi classification) or as stages ranging from 1 to 4 (as per Gass and Blodi classification).

**Table 2:** Optical coherence tomography (OCT) features noted in different clinical stages of type 2 MacTel identified on retinal examination

Classification	Gass and Blodi [2]					Yannuzzi et al. [3]	
Clinical stages	Loss of retinal transparency and presence of perifoveal greying (Stage 1) (N = 31)	No or occult visible telangiectasias (Stage 2) (N = 35)	Dilated right angled venules (Stage 3) (N = 73)	Retinal pigment plaques (Stage 4) (N = 38)	Presence of SRNVM or proliferative disease (Stage 5) (N = 35)	Non-proliferative stage (N = 175)	Proliferative stage (N = 37)
SDOCT features							
Irregularity of foveal contour (N, %)	2 (6)	4 (11)	12 (16)	22 (58)	26 (74)	39 (22)	27 (73)
ILM drape (N, %)	12 (39)	17 (49)	27 (37)	2 (5)	4 (11)	57 (33)	5 (14)
Hyperreflective middle retinal layers (N, %)	30 (97)	34 (97)	69 (95)	35 (92)	16 (46)	166 (95)	18 (49)
Superficial hyperreflective retinal dots (N, %)	0 (0)	0 (0)	1 (1)	3 (8)	2 (6)	4 (2)	2 (5)
Hypo reflective inner retinal cavities (N, %)	16 (52)	26 (74)	39 (53)	13 (34)	10 (29)	93 (53)	11 (30)
Outward bending of inner retinal layers (N, %)	7 (23)	0 (0)	29 (40)	20 (53)	8 (23)	66 (38)	8 (22)
Hypo reflective outer retinal cavities (N, %)	7 (23)	9 (26)	13 (18)	3 (8)	0 (0)	32 (18)	0 (0)
Hyperreflective retinal pigment clumps (N, %)	1 (3)	3 (9)	11 (15)	37 (97)	22 (63)	52 (30)	22 (59)
Subfoveal SRF (N, %)	2 (6)	3 (9)	18 (25)	2 (5)	9 (26)	24 (14)	10 (27)
SRNVM (N, %)	1 (3)	2 (6)	10 (14)	3 (8)	25 (71)	15 (9)	25 (70)
RCA (N, %)	1 (3)	1 (3)	2 (3)	5 (13)	10 (29)	10 (6)	10 (27)

ILM, internal limiting membrane; SRNVM, subretinal neovascular membrane; RCA, retino-choroidal anastomosis; SRF, subretinal fluid

For further understanding, the eyes were divided into two groups on the basis of SDOCT findings of proliferation (SRNVM/RCA): Group 1: Non-proliferative type 2 MacTel and Group 2: Proliferative type 2 Mactel. Comparative SDOCT features between the two groups is described in Table 3. Presence of hyperreflective MRL (p < 0.001), hyporeflective inner (p = 0.032) and outer retinal (p = 0.002) cavities and ILM drape (p = 0.031) were commonly associated with poor vision in the non-proliferative group. The proliferative stage of the disease showed higher prevalence of hyperreflective RPC (p = 0.002), subfoveal SRF (p = 0.037) and foveal contour irregularity (p < 0.001). No significant difference was noted between the two groups for superficial retinal crystals (p = 0.055) and outward bending of IRL (p = 0.873).

**Table 3:** Comparison of the different SDOCT imaging features between the non-proliferative and proliferative type 2 MacTel groups

Variable	Non-proliferative (n = 152)	Proliferative (n = 60)	P value
Age	62.2 ± 9.48	62.2 ± 9.38	0.967
LogMAR VA	0.389 ± 0.316	0.596 ± 0.335	< 0.001
Irregularity of foveal contour (N, %)	35 (23)	31 (52)	< 0.001
ILM drape (N, %)	51 (34)	11 (18)	0.031
Hyperreflective middle retinal layers (N, %)	148 (97)	36 (60)	< 0.001
Superficial hyperreflective retinal dots (N, %)	2 (1)	4 (6)	0.055
Hypo reflective inner retinal cavities (N, %)	82 (54)	22 (37)	0.032
Outward bending of inner retinal layers (N, %)	54 (36)	20 (33)	0.873
Hypo reflective outer retinal cavities (N, %)	30 (20)	2 (3)	0.002
Hyperreflective retinal pigment clumps (N, %)	43 (28)	31 (52)	0.002
Subfoveal SRF (N, %)	19 (12)	15 (25)	0.037

VA, visual acuity; ILM, internal limiting membrane; SRF, subretinal fluid

### Correlation between OCT features and visual acuity

Correlations between the SDOCT imaging features and visual acuity in the whole dataset and also between the two groups were studied using the Spearman’s correlation test. Poor vision was noted with the presence of foveal contour irregularity (r = 0.289; p < 0.001), hyperreflective RPC (r = 0.294; p < 0.001), SRNVM (r = 0.242; p < 0.001) and RCA (r = 0.137; p = 0.046) (Table 4) in patients with type 2 MacTel. Multiple linear regression analyses were performed to identify the SDOCT features showing the best correlations with the visual acuity. Presence of hyperreflective RPC (p = 0.012) and SRNVM (p = 0.004) showed statistically significant changes with vision. Foveal contour irregularity (r = 0.197; p = 0.01), outward turning of IRL (r = 0.252; p = 0.002) and hyperreflective RPC (r = 0.244; p = 0.002) were identified as the factors which correlated with poor vision in the non-proliferative group (Table 5). Likewise, poor vision was noted with presence of foveal irregularity (r = 0.28; p = 0.03) and RPC (r = 0.255; p = 0.048) in the proliferative group (Table 6).

**Table 4:** Correlation between the different SDOCT imaging features and visual acuity (logMAR) using the Spearman’s correlation test for the whole dataset

LogMAR VA with	r value	P (two-tailed)	95% confidence interval
Foveal irregularity	0.289	< 0.001	0.157 to 0.411
ILM drape	− 0.039	0.57	− 0.177 to 0.100
Hyperreflective middle retinal layers	− 0.269	0.382	− 0.393 to − 0.135
Superficial hyperreflective retinal dots	0.074	0.28	− 0.065 to 0.211
Hypo reflective inner retinal cavities	− 0.106	0.12	− 0.241 to 0.033
Outward turning of inner retinal layers	0.126	0.07	− 0.013 to 0.260
Hypo reflective outer retinal cavities	− 0.094	0.17	− 0.229 to 0.045
Sub foveal SRF	0.116	0.09	− 0.023 to 0.250
Hyperreflective retinal pigment clumps	0.294	< 0.001	0.162 to 0.416
SRNVM	0.242	< 0.001	0.107 to 0.368
RCA	0.137	0.046	− 0.001 to 0.271

ILM, internal limiting membrane; SRF, subretinal fluid; SRNVM, sub retinal neovascular membrane; RCA, retino-choroidal anastomosis; VA, visual acuity

**Table 5:** Correlation between the different SDOCT imaging features and visual acuity (logMAR) in the non-proliferative group using the Spearman’s correlation test

LogMAR VA with	r	P (two-tailed)	95% confidence interval
Foveal irregularity	0.197	0.01	0.034 to 0.350
ILM drape	− 0.0297	0.72	− 0.193 to 0.135
Hyperreflective middle retinal layers	− 0.129	0.11	− 0.286 to 0.036
Superficial hyperreflective retinal dots	0.0174	0.83	− 0.147 to 0.181
Hypo reflective inner retinal cavities	− 0.0687	0.4	− 0.230 to 0.096
Outward turning of inner retinal layers	0.252	0.002	0.092 to 0.400
Hypo reflective outer retinal cavities	0.003	0.98	− 0.161 to 0.166
Sub foveal SRF	0.0964	0.24	− 0.069 to 0.256
Hyperreflective retinal pigment clumps	0.244	0.002	0.084 to 0.392

ILM, internal limiting membrane; SRNVM, sub retinal neovascular membrane; RCA, retino-choroidal anastomosis; VA, visual acuity; SRF, subretinal fluid

**Table 6:** Correlation between the different SDOCT imaging features and visual acuity (logMAR) in the proliferative group using the Spearman’s correlation test

LogMAR VA with	r	P (two-tailed)	95% confidence interval
Foveal irregularity	0.288	0.03	0.029 to 0.511
ILM drape	0.13	0.32	− 0.136 to 0.378
Hyperreflective middle retinal layers	− 0.184	0.16	− 0.425 to 0.081
Superficial hyperreflective retinal dots	0.043	0.74	− 0.221 to 0.301
Hypo reflective inner retinal cavities	− 0.038	0.78	− 0.296 to 0.226
Outward turning of inner retinal layers	− 0.16	0.22	− 0.405 to 0.105
Hypo reflective outer retinal cavities	− 0.266	0.067	− 0.493 to − 0.006
Sub foveal SRF	− 0.007	0.96	− 0.267 to 0.255
Hyperreflective retinal pigment clumps	0.255	0.048	− 0.007 to 0.484

ILM, internal limiting membrane; SRNVM, sub retinal neovascular membrane; RCA, retino-choroidal anastomosis; VA, visual acuity; SRF, subretinal fluid

## Discussion

The current study summarizes the characteristic imaging findings of type 2 Mactel on SDOCT and its relationship with clinical stages and visual acuity. A combination of findings could be identified with the hyperreflective MRL being the most common one. Presence of foveal contour irregularity, hyporeflective degenerative outer retinal cavities, hyperreflective RPC and presence of subfoveal SRF were commonly noted with the proliferative stages. Certain features on SDOCT like foveal contour irregularity, outward turning of IRL and hyperreflective RPC were associated with poor vision in the non-proliferative group. Likewise, poor vision was noted with presence of foveal irregularity and RPC in the proliferative group.

Hyperreflective MRL has been identified as one of the earliest findings of type 2 MacTel. This has been noted due to increased capillary leakage [1]. Another theory for the increased hyperreflectivity of the MRL could be the loss of the Müller cells in the perifoveal area. There are a number of studies in literature to suggest that the Müller cells provide structural stability to the fovea and they form a part of the inner blood retinal barrier [9, 10]. In type 2 Mactel, there is loss of perifoveal Müller cells [11, 12]. This exposes the deep retinal capillaries and makes them easily visible on SDOCT as hyperreflective lesions in the MRL. In our study, we noted the hyperreflective MRL in the perifoveal region (87%) as the most common SDOCT finding. In the early disease stages, this needs to be differentiated from disorganised retinal inner layers where there is loss of IRL stratification. In type 2 MacTel, the retinal layers stratification in the MRL seems to be maintained. Thus, it would be appropriate to hypothesize that hyperreflective MRL is an early sign seen on SDOCT in type 2 MacTel. However, with the further progression of the disease into the proliferative stage, hyperreflectivity gets reduced possibly due to loss of supporting structures, surrounding atrophy and presence of retinal or subretinal neovascularisation.

Hyporeflective cavities in inner retina was the second most common finding noted in the current study. These cavities are usually degenerative, resulting from the loss of structural integrity and there is no angiographic leakage and pooling of fluorescein dye into these hyporeflective spaces as suggested by Koizumi et al. [13] We noted the prevalence of hyporeflective inner retinal cavity was reduced in the proliferative stage of the disease. This also could possibly be due to the same reasons as mentioned above.

Other less prevalent inner retinal OCT findings noted in our study included the irregularity of the foveal floor and ILM drape sign. Though the foveal asymmetry is a much earlier sign seen in asymptomatic cases of type 2 MacTel, it was not frequently seen in our study cohort. This minor structural alteration seen in the foveal contour occurs due to the changes in the outer nuclear/Henle’s fibre layer thickness [1, 14]. However, if capillary leakage occurs within the same area as seen in the hyperreflective MRL, this asymmetric foveal contour may disappear due to a slight thickening mainly within the IRL [1]. As most of the cases in our series already presented with an hyperreflective MRL, this subtle finding of foveal contour asymmetry became less prevalent on SDOCT. Also, in proliferative disease, the presence of neovascularisation or intraretinal fluid causes IRL thickening and thereby making the foveal contour asymmetry less prominent. ILM drape sign is an important OCT characteristic of type 2 MacTel. The ILM drape sign occurs when a thin membrane overhangs a central cystoid lesion at the base of the fovea of normal contour and thickness. In our study, ILM drape sign was seen in 34% cases in the non-proliferative group and in only 18% cases in the proliferative group. This lower prevalence in the proliferative group could be explained due to the masking of the ILM drape sign by the presence of the subfoveal SRF and presence of SRNVM/RCA. This similar masking effect has been previously noted in a report by Abdul-Rahman, where the ILM drape sign showed up again following the resolution of the subfoveal SRF after treatment with intravitreal Bevacizumab injections [15].

Superficial intraretinal crystals are a frequent clinical finding associated with type 2 MacTel [16]. Their presence supports in the early disease diagnosis and they are present in all stages. Their morphology further implicates the Müller cells in the type 2 MacTel pathogenesis [16]. However, on SDOCT, fine superficial hyperreflective retinal dots are less frequently identified. In the current study, only 3% cases showed the retinal crystals.

Most frequent changes in the outer retina on SDOCT noted in our study included outward turning of IRL, presence of migrated RPC, subfoveal SRF and presence of hyporeflective outer retina cavities. The turning of IRL towards the outward retina occurs due to the collapse of the outer retinal layers with co-existing damage to the ELM and ellipsoid zone layers. The presence of this characteristic SDOCT finding in the non-proliferative group (r = 0.252; p = 0.002) was associated with significantly poor vision. Similar observations regarding the collapse of the outer retinal layers and visual acuity were noted by Kim et al. [6] Hyperreflective intraretinal lesions are usually associated with pigment plaques. Over all the retinal pigment plaques were noted in 35% of cases in the current study and were more commonly seen in the proliferative group (52%). The presence of the RPC was associated significantly with reduced visual acuity in both non-proliferative (r = 0.244; p = 0.002) and proliferative stages (r = 0.255; p = 0.048) of the disease. In  > 50% of all proliferative cases in our study, the neovascularisation coincided with the RPC. Thus, the presence of RPC could act as a precursor or biomarker for suspecting the proliferative stage of the disease. Leung et al. noted similar observations in their study and suggested refining of the current staging of type 2 MacTel based on the pigment plaque characteristics [17].

The presence of outer retinal hyporeflective cavities was more commonly encountered in the non-proliferative disease stages compared to the proliferative group. These occur due to tissue loss in the photoreceptor layer. In our study, we noted subfoveal SRF in 34 eyes, of which 15 eyes were associated with proliferative disease. In 19 eyes, the subfoveal detachment was noted in the non-proliferative stages of the disease. Manayath et al. reported 20 eyes in 13 patients with subfoveal detachment in non-proliferative type 2 MacTel [18]. There was reduction in the SRF in all cases with improvement in vision by > 1-line in 7 of the 8 eyes following intravitreal anti-VEGF injections. The existing classification by Gass and Blodi or that by Yannuzzi et al. does not allow accurate categorisation of this disease stage [2, 3]. In the current cohort, 60 eyes with proliferative disease were identified. Most of them were SRNVM (68%) while the remaining showed RCA (32%).

Our study provides an understanding of the different SDOCT features, its correlation with the clinical staging and visual outcome (Table 7) which can assist the ophthalmologist in suspecting proliferative disease and plan the management and follow-up visit accordingly. Eyes with suspected proliferation can be confirmed by using non-invasive imaging techniques like OCT-angiography. This limits the use of unnecessary invasive tests like fluorescein angiography for the diagnosis of proliferative stage of type 2 MacTel.

**Table 7:** Correlation between the spectral domain optical coherence tomography (SDOCT) features, clinical staging and visual acuities in type 2 MacTel

No	SDOCT features	Frequency	Clinical stage	Visual prognosis
1	Hyperreflective middle retinal layers	Very common	Non-proliferative disease	Good
2	Hypo reflective inner retinal cavities	Common	Non-proliferative disease	Good
3	ILM drape	Common	Non-proliferative disease	Good
4	Irregularity of foveal contour	Less common	Non-proliferative disease	Good
5	Superficial hyperreflective retinal dots	Uncommon	Non-proliferative disease	Good
6	Hypo reflective outer retinal cavities	Common	Non-proliferative disease	Good
7	Outward bending of IRL without subfoveal SRF	Common	Suspect proliferative disease	Poor
8	Outward bending of IRL with subfoveal SRF	Less common	Suspect proliferative disease	Poor
9	Retinal pigment clump	Less common	Suspect proliferative disease	Poor
10	SRNVM/RCA	Less common	Proliferative disease	Poor
11	Irregularity of foveal contour along with SRNVM/RCA	Common	Proliferative disease	Poor

Good = vision ≥ 20/50 and Poor = vision < 20/50

ILM, internal limiting membrane; IRL, inner retinal layers; SRF, subretinal fluid; SRNVM, subretinal neovascular membrane; RCA, retinochoroidal anastomosis

The major drawback of this study is its retrospective analysis of SDOCT images at the baseline visit without analysis of the longitudinal data. Also, we assessed the visual acuity as the only functional outcome in this study even when other functional measures such as near vision, microperimetry changes and multifocal electroretinography changes would have been more conclusive. Also, we did not investigate the other outer retinal findings such as discontinuity of the ELM and ellipsoid zone and correlate with visual acuity. In the presence of RPC and SRNVM, accurate detection of these changes would have been difficult; hence, we did not analyse these features in this study. The strengths are the large cohort of cases from a single centre, imaged on the same SDOCT machine and analysed by a single observer, thereby providing consistency in the imaging findings across the different disease stages.

In conclusion, the above summarised SDOCT imaging features help in diagnosing and staging type 2 MacTel. Presence of hyperreflective RPC and SRNVM showed significantly poor vision. Additional prospective long-term studies can help to describe the progressive and sequential nature of structural changes and the disease course.

## Data Availability

The datasets used and/or analysed during the current study are available from the corresponding author on reasonable request.

## Abbreviations

MacTel: Macular telangiectasia
ILM: Internal limiting membrane
SDOCT: Spectral domain optical coherence tomography
RPC: Retinal pigment clumps
SRNVM: Subretinal neovascular membrane
MRL: Middle retinal layers
IRL: Inner retinal layers
SRF: Subretinal fluid
RCA: Retinochoroidal anastomosis
ELM: External limiting membrane
RPE: Retinal pigment epithelium
